# Giant ileal inflammatory fibroid polyp causing small bowel obstruction: a case report and review of the literature

**DOI:** 10.1186/1757-1626-1-341

**Published:** 2008-11-21

**Authors:** Sagal O Mohamud, Shahina A Motorwala, AM Rebecca Daniel, Joseph A Tworek, Thomas M Shehab

**Affiliations:** 1 Department of Internal Medicine, Saint Joseph Mercy Hospital, Ypsilanti, MI, USA; 2 Huron Gastro, Ypsilanti, MI, USA; 3 Department of Pathology, Saint Joseph Mercy Hospital, Ypsilanti, MI, USA

## Abstract

**Introduction:**

There are several types of small bowel pathology that can lead to small bowel obstruction or intussusception. The etiology causing small bowel obstruction varies by age. Benign disease is the typical cause in children and adolescents while malignant or adhesive disease is far more common in older patients. Although cases of adult intussusception caused by benign processes are rare, there are reports of inflammatory fibroid polyps causing adult intussusception of the terminal ileum published in the literature.

**Case presentation:**

We present the case of a 70-year-old man with a multiple year history of intermittent episodes of bowel obstruction who was found to have a giant ileal inflammatory fibroid polyp causing intermittent small bowel obstruction. The patient underwent operative intervention and has now been symptom-free for three years.

**Conclusion:**

Small bowel lesions include both malignant and benign etiologies. The malignant etiologies include adenocarcinoma, carcinoid or lymphoma while benign lesions are typically lipomas, inflammatory polyps or adenomas. Inflammatory fibroid polyps are rare, benign lesions that can occur anywhere within the gastrointestinal tract. They are typically an incidental finding, but on rare occasions have been presented as the source of intussusception or obstruction.

## Introduction

There are several types of small bowel pathology that can lead to small bowel obstruction or intussusception. The etiology causing small bowel obstruction varies by age. Benign disease is the typical cause in children and adolescents while malignant or adhesive disease is far more common in older patients [1,2]. Although cases of adult intussusception caused by benign processes are rare, there are reports of inflammatory fibroid polyps causing adult intussusception in the terminal ileum published in the literature.

## Case presentation

A 70-year-old gentleman presented for gastroenterology consultation after multiple episodes of severe abdominal pain that occurred over a period of six years. The patient had a past medical history of hypothyroidism and hypertension. He reported several episodes of nearly identical GI symptoms over this period of time. The episodes would begin with right upper quadrant abdominal pain that would be relatively sudden in onset and 3 out of 10 in severity. Over a matter of hours, the pain would crescendo to 10 out of 10 and would last three to four additional hours. During this period of time, the patient would pass very little flatus and would develop abdominal distention, severe diaphoresis, and nausea. The pain would settle in the right lower quadrant for several hours and then would almost completely resolve. The patient would then remain "sore" for a period of one to two additional days. On a few occasions, he had vomiting. He had two such episodes with vomiting prior to presenting for evaluation.

Between the episodes, the patient reported normal bowel habits. He had no history of diarrhea, weight loss, or significant nausea or vomiting. The patient had no history of anemia or GI bleeding. He had never undergone a colonoscopy in the past. The patient did not use aspirin or other non-steroidal anti-inflammatory medications on a regular basis.

The patient's physical exam was unremarkable. The patient was afebrile, vital signs were stable. The patient's abdomen was soft, non-tender and non-distended. There were no palpable masses in abdomen. There were no incisional scars on the abdomen.

Evaluation included a CT scan of the abdomen and pelvis. The CT scan showed a benign appearing renal cyst and an area of abnormal ileum. An approximately 12 centimeter long portion of ileum demonstrated concentric thickening. The radiologist offered a presumptive diagnosis of Crohn's.

**Figure 1:**
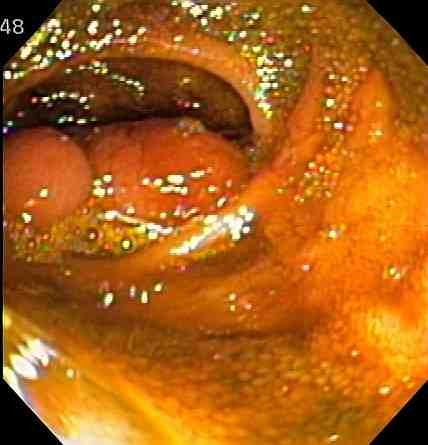
Endoscopy image of a pedunculated polyp filling ileal lumen.

Laboratory evaluation demonstrated a normal complete blood count and normal iron studies. The patient underwent colonoscopy to further evaluate the ileal thickening. He tolerated colonoscopy prep without difficulty. Colonoscopy demonstrated left colon diverticula. The terminal ileum was abnormal. The mucosa appeared atrophic and the luminal diameter was narrowed. There was also a mobile, pedunculated 3.5 centimeter frond-like mass. During the endoscopic evaluation, this lesion was seen to move proximally and distally over a span of at least 5 to 7 cm. It was difficult to biopsy given its movement. When the mass moved distally it filled the entire terminal ileal diameter (Figure 1). The mass moved to the area of the IC valve but was not seen to enter the colon. Biopsies of the terminal ileum and the ileal mass showed scattered ulcers with crypt distortion and pyloric gland metaplasia suggestive of Crohn's disease. This did not explain the presence of a mass. Surgical management was recommended but the patient refused.

**Figure 2:**
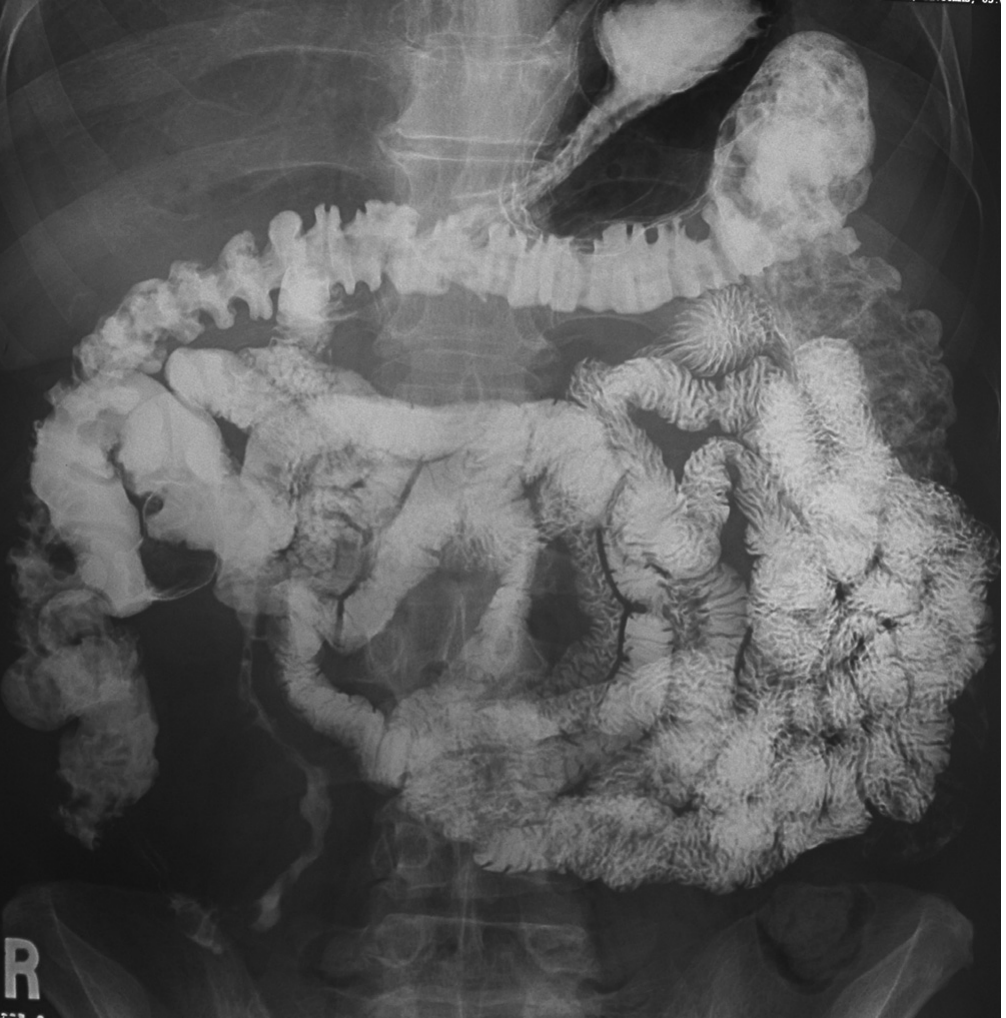
X-ray demonstrating 18 cm of abnormal small bowel in the distal ileum with effacement of the small bowel folds.

The patient had two additional episodes of abdominal pain in the months after the colonoscopy. After one of the episodes he underwent a small-bowel follow through which demonstrated 18 cm of abnormal small bowel in the distal ileum with effacement of the small bowel folds (Figure 2). No mention of the ileal mass was made on the small bowel follow through.

**Figure 3:**
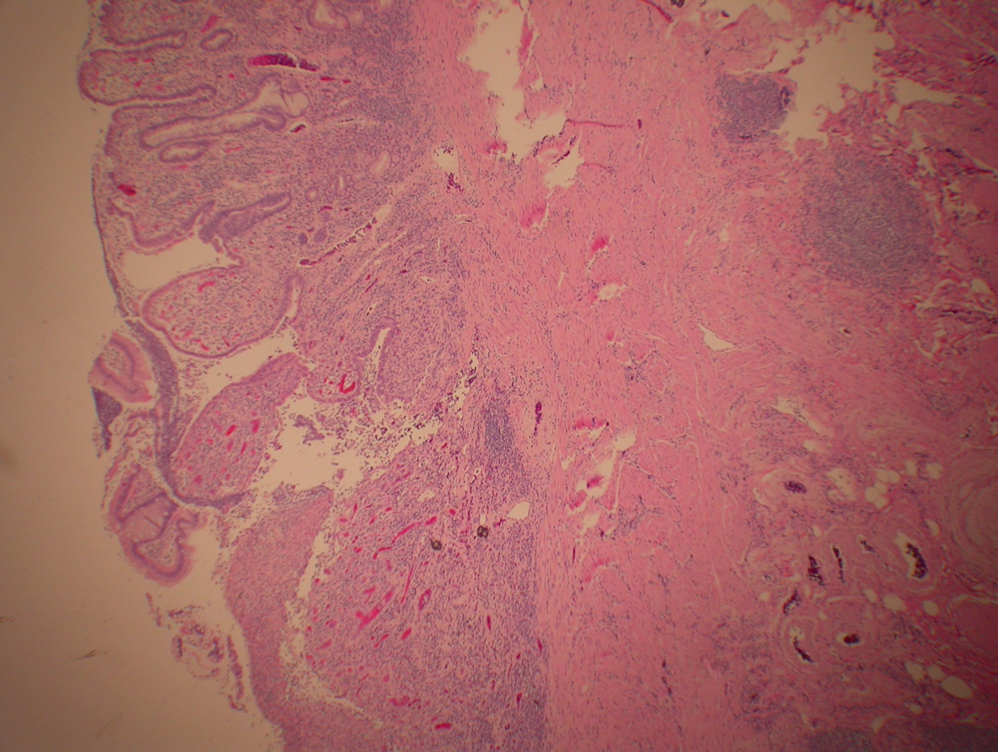
**Inflammatory fibroid polyp projecting into lumen of terminal ileum.** The polyp is covered by flattened ileal mucosa containing distorted crypts with branching. The polyp contains a broad stalk containing fibroblasts, eosinophils, dilated blood vessels and nodules of lymphocytes (hematoxylin-eosin, original magnification × 20).

**Figure 4:**
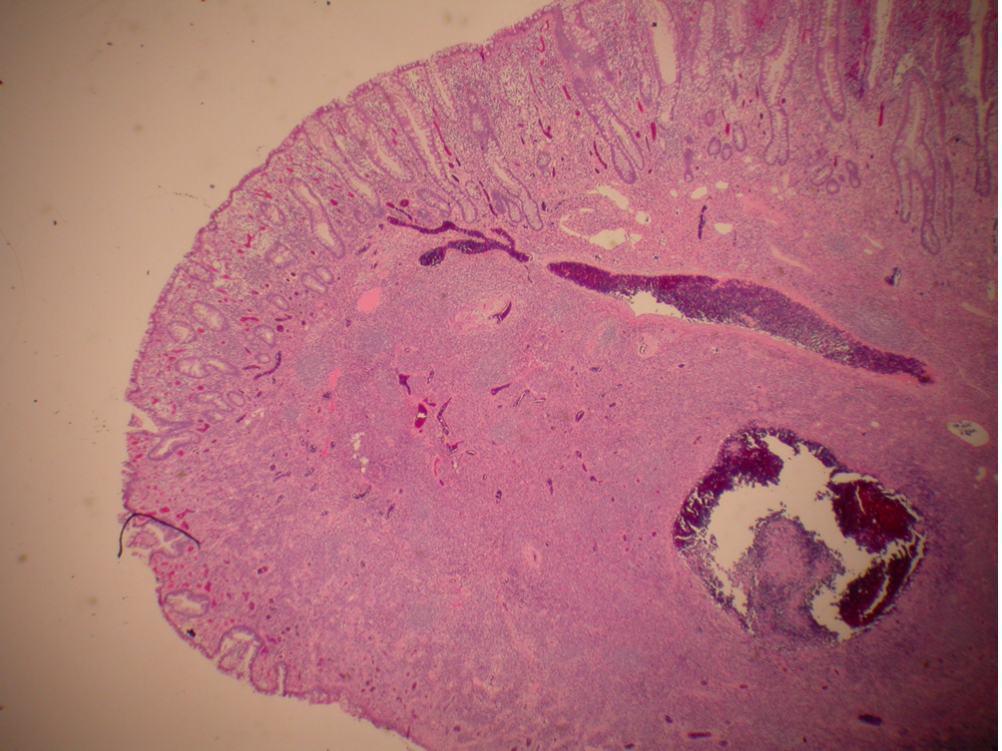
Terminal ileum just proximal to inflammatory fibroid polyp displays a crohns-like reaction pattern with ulceration, crypt distortion, pyloric gland metaplasia and submucosal lymphocytic inflammation (hematoxylin-eosin, original magnification × 40).

The patient then agreed to surgical evaluation and underwent laparoscopic assisted right hemicolectomy. Exploratory laparotomy showed a thickened portion of ileum. Pathologic exam showed a 3 cm inflammatory fibroid polyp composed of an edematous stroma containing spindle shaped stromal cells, lymphoid nodules and eosinophils (Figure 3). There was an adjacent "Crohn's like" inflammatory reaction similar to that seen in the initial biopsies. Granulomas were not identified (Figure 4).

The patient has been symptom free for more than three years. He has had no further obstructive symptoms and he has no symptoms suggesting Crohn's disease.

## Conclusion

Bowel obstruction can occur as a result of a mechanical blockage or dysfunction of the small or large intestine. Only 5% of intestinal invaginations or intussusceptions occur in adults. Cases of adult intussusceptions account for only 1–5% of all small bowel obstructions [2]. Patients with intussusception usually present with abdominal pain and nausea. Pre-operative diagnosis of intussusception is rare but can occur in finding a palpable mass on the abdomen or with the use of imaging techniques. The majority of intussusceptions, however, are diagnosed during operation [3].

Sixty-five percent of all adult intussusceptions occur due to a malignant or benign lesion usually appearing at the head of the invagination. The probability of malignancy is greater for those cases occurring in the large intestine. Malignant lesions are found in 43% to 80% of intussusceptions located in the colon compared to only about 14% to 47% of cases occurring in the small intestine. Benign tumors as the lead points of an intussusception have been found to include lipomas, leiomyomas, neurofibromas, adenomas, and inflammatory fibroid polyps [2].

The underlying cause of Inflammatory fibroid polyps (IFPs) is still unknown. Many have suggested etiologies possibly related to chemical, physical, or metabolic triggers [4]. IFPs were first described in the literature as "gastric submucosal granuloma with eosinophilic infiltration" in a study by Vanek in 1949. They later became recognized under a variety of different names: inflammatory pseudotumor, granuloma with eosinophils, eosinophilic granuloma, and polyp with eosinophilic granuloma. Four years after their initial appearance in the literature, the term inflammatory fibroid polyp was introduced in a study by Helwig et al. and has since become the most widely used [5].

In a case series of 75 patients presenting with inflammatory fibroid polyps, Johnstone et al. reported three-fourths of all cases as gastric IFPs [6]. The occurrences of inflammatory fibroid polyps in the small intestine are rare, accounting for only 18% of all cases [6]. This case is one of the few reported in the literature where intussusception is caused by a terminal ileum inflammatory fibroid polyp, Table 1[3,7-11]. Cases of intussusception in the elderly resulting from IFPs are also especially rare.

**Table 1:** Clinical Presentation, Endoscopic findings, and Characteristics of Inflammatory Fibroid Polyps of the Terminal Ileum Causing Adult Intussusception in the English Literature

**Case**	**Age**	**Gender**	**Symptoms**	**Evaluation**	**# of IFP(s)**	**Size of IFP (cm)**	**Other Findings**	**Reference**
1	47	M	unexplained diarrhea, colicky abdominal pain, and moderate nausea	Physical examination, laboratory investigations, colonoscopy, laparotomy, hemicolectomy	2	N/A	N/A	3

2	51	M	Crampy periumbilical pain and nausea	3 radiologic views of abdomen, contrast barium enema refluxed, laparotomy, small bowel resection with primary anastomosis	1	2.8 × 4 × 4	Surface of polyp ulcerated, focal mucosal ulceration on small-bowel wall opposing the mass	7

3	35	F	Diffused abdominal pain, distention of the abdomen, inability to pass flatus and stools, diarrhea, and weight loss	Physical examination, abdominal ex-ray, laboratory investigations, Emergency laparotomy, small bowel enterectomy and end-to-end anastomosis	1	4	Crohn's disease	8

4	84	F	Abdominal pain and weight loss	Examination, Ultrasound, small bowel barium meal, laboratory investigations, and laparotomy	1	7 × 3.5 × 4.5	Polyp covered by ulcerated mucosa, active Crohn's disease	9

5	70	M	Perforated duodenal ulcer, passing blood clots and mucus per rectum, and paroxysmal, cramp-like central abdominal pain	Physical examination, plain abdominal radiograph, emergency laparotomy, and right hemicolectomy	1	2 × 1.3 × 1.1	N/A	10

6	59	F	N/A	N/A	1	3	Surface erosion or ulceration on polyp	11

8	70	M	Intermittent bowel obstruction, little flatus, abdominal distention, severe diaphoresis, and nausea	Physical examination, CT Scan, laboratory investigations, Colonoscopy, x-ray, laparoscopic assisted right hemicolectomy	1	3	Surface of polyp ulcerated, Crohn's-like reaction	*

*Current Case

Cases of intussusception have been reported secondary to Crohn's disease [12]. While this was a consideration in our case, the Crohn's like inflammatory response is consistent with chronic injury from the inflammatory fibroid polyp. This is also supported by the lack of granulomatous inflammation in the excision specimen and the patient's asymptomatic course since excision.

## Abbreviations

IFP: Inflammatory Fibroid Polyp

## Consent

Written informed consent was obtained from the patient for publication of this case report and accompanying images. A copy of the written consent is available for review by the Editor-in-Chief of this journal.

## Competing interests

The authors declare that they have no competing interests.

## Authors' contributions

SOM was involved in the drafting of the article, critical revision of the article for important intellectual content. SAM was involved in the conception and design. AMR was involved in the conception and design. JAT was involved in the drafting of the article, analysis and interpretation of the data, critical revision of the article for important intellectual content. TMS was involved in the conception and design, drafting of the article, analysis and interpretation of the data, critical revision of the article for important intellectual content. All authors contributed in the final approval of the article.
